# Lifestyle Behaviours and Antiplatelet Medication Adherence Among Post-Percutaneous Coronary Intervention Patients in Saudi Arabia: Implications for Holistic Cardiovascular Nursing Care

**DOI:** 10.3390/healthcare14070914

**Published:** 2026-04-01

**Authors:** Muteb Aljuhani, Rayhanah R. Almutairi, Waleed M. Alshehri, Abdulaziz M. Alodhailah

**Affiliations:** 1 Department of Community Health, Mental and Psychiatric Nursing, Imam Mohammad Ibn Saud Islamic University (IMSIU), Riyadh 11564, Saudi Arabia; 2 Community and Psychiatric Mental Health Nursing Department, College of Nursing, Princess Nourah bint Abdulrahman University, Riyadh 11671, Saudi Arabia; 3 Department of Medical-Surgical Nursing, College of Nursing, King Saud University, Riyadh 11451, Saudi Arabia

**Keywords:** lifestyle behaviours, smoking, physical activity, medication adherence, percutaneous coronary intervention, cardiovascular nursing, Saudi Arabia, secondary prevention

## Abstract

**Background:** Lifestyle behaviours and medication adherence are interrelated components of cardiovascular secondary prevention, yet their co-occurrence in Middle Eastern post-percutaneous coronary intervention (PCI) populations remains poorly characterised. This study described smoking status and physical activity patterns, assessed antiplatelet medication adherence, and explored associations between lifestyle factors and adherence among Saudi patients following PCI. **Methods:** A cross-sectional survey was conducted among 236 Saudi adults who had undergone PCI within the preceding 12 months at two tertiary cardiac centres in Riyadh, Saudi Arabia. Data were collected on smoking status, cigarette consumption, self-reported physical activity frequency (defined as the frequency of engagement in regular exercise such as walking, swimming, or other structured physical activity), and self-reported medication adherence measured via the Morisky Medication Adherence Scale-8 (MMAS-8). Descriptive statistics characterised lifestyle and adherence patterns. Bivariate analyses (chi-square tests) and multivariate binary logistic regression were used to explore associations between lifestyle factors and adherence, adjusting for comorbidities including hypertension, diabetes mellitus, arthritis, and heart disease. **Results:** Participants were predominantly male (73.7%) and older adults (83.9% aged >50 years). Smoking prevalence was 23.3% (*n* = 55 of 236 participants), and physical inactivity was highly prevalent, with 57.2% of the sample (*n* = 135) reporting never engaging in regular exercise. Adherence was suboptimal, with 55.4% of participants (*n* = 129) classified as having low adherence (self-reported, measured via the MMAS-8). In multivariate analysis, arthritis was the only statistically significant predictor of adherence (adjusted odds ratio [AOR] = 2.81, 95% confidence interval [CI]: 1.01–7.84, *p* = 0.048; note, however, that this finding does not survive Bonferroni correction for multiple comparisons and should be interpreted as hypothesis-generating). Smoking (AOR = 0.52, 95% CI: 0.19–1.45, *p* = 0.213) and physical inactivity (AOR = 0.45, 95% CI: 0.09–2.25, *p* = 0.332) showed inverse but statistically non-significant trends with adherence. **Conclusions:** Unhealthy lifestyle behaviours and low medication adherence were each highly prevalent and co-occurred in this post-PCI population, though associations between lifestyle factors and adherence were not statistically confirmed except for arthritis. These descriptive findings are hypothesis-generating and provide a rationale for future adequately powered prospective studies and trials evaluating integrated nursing interventions that simultaneously address smoking, physical inactivity, and medication non-adherence in Saudi cardiac care settings.

## 1. Introduction

Cardiovascular diseases (CVDs) remain the foremost cause of mortality worldwide, accounting for approximately 17.9 million deaths annually, with prevalence projected to increase as populations age and risk factors such as diabetes, hypertension, and obesity continue to rise [1]. Among the diverse therapeutic modalities for coronary artery disease, percutaneous coronary intervention (PCI) has emerged as a cornerstone, offering effective revascularisation, reduced hospital stay, and improved quality of life [2,3]. The long-term clinical success of PCI, however, is contingent upon strict adherence to secondary prevention regimens, particularly dual antiplatelet therapy (DAPT), which is essential for preventing stent thrombosis, recurrent myocardial infarction, and cardiovascular death [4,5].

Cardiovascular secondary prevention encompasses multiple interrelated health behaviours, including medication adherence, smoking cessation, regular physical activity, and dietary modification [6]. Following PCI, patients are advised to adopt comprehensive lifestyle changes alongside prescribed antiplatelet therapy to minimise the risk of recurrent cardiovascular events [7,8]. However, adherence to these recommendations is often suboptimal, with many patients struggling to sustain healthy behaviours over time [9].

A growing body of evidence indicates that health behaviours tend to cluster: individuals who engage in one unhealthy behavior, such as smoking are more likely to exhibit other risk behaviours, including physical inactivity and poor medication adherence [10,11]. This clustering phenomenon has important implications for intervention design, as addressing behaviours in isolation may be less effective than comprehensive approaches targeting multiple risk factors simultaneously [12]. Globally, medication adherence has been described as a complex, multidimensional phenomenon shaped by patient, provider, and health system-level factors [13,14,15,16]. Theoretical models such as the Theory of Planned Behaviour (TPB) and the Capability, Opportunity, and Motivation–Behaviour (COM-B) model have been applied to elucidate behavioural determinants of adherence, emphasising the role of attitudes toward medication, perceived social norms, and patients’ sense of control [17,18].

Smoking remains a modifiable cardiovascular risk factor that warrants particular attention following PCI. Continued smoking after coronary revascularisation substantially increases the risk of stent thrombosis, recurrent myocardial infarction, and cardiovascular mortality [19]. Moreover, smoking has been associated with poor medication adherence across multiple chronic conditions, potentially reflecting shared psychological and behavioural determinants such as low health consciousness and reduced self-efficacy [20,21].

Regular physical activity is similarly important for cardiovascular secondary prevention. Regular exercise improves cardiac function, enhances quality of life, and reduces all-cause mortality following PCI [22]. Structured cardiac rehabilitation programmes incorporating supervised exercise have demonstrated benefits for both physical outcomes and medication adherence [23]. Conversely, sedentary behaviour has been linked to reduced engagement in health-promoting activities, including medication-taking [24].

In Saudi Arabia, cardiovascular disease represents a leading cause of morbidity and mortality, driven by high prevalence of diabetes, hypertension, and obesity [25]. Lifestyle factors contribute substantially to this burden: smoking rates among Saudi adults remain concerning despite public health efforts, and physical inactivity is widespread, particularly among older adults and those with chronic conditions [26]. In the Middle Eastern region, adherence to antiplatelet and other cardiovascular medications following coronary interventions is particularly challenging. A systematic review of heart failure medication adherence across Arab countries revealed non-adherence rates of up to 60%, with financial constraints, cultural beliefs regarding fate, and insufficient health literacy as prominent barriers [27].

Recent research on medication literacy among Saudi adults revealed that nearly half scored below average, indicating significant vulnerability to mismanagement of long-term pharmacotherapy [28]. Understanding how lifestyle patterns relate to medication adherence in post-PCI patients can inform the development of culturally appropriate, comprehensive secondary prevention nursing programmes.

Nurses play a central role in cardiovascular secondary prevention, delivering education, counselling, and support across the care continuum [29]. Systematic reviews demonstrate that nurse-led counselling, motivational interviewing, and follow-up interventions are effective in improving adherence across chronic conditions [30]. In cardiac populations, interventions that integrate education with behavioural support have been shown to significantly enhance adherence and reduce readmissions [30]. However, successful implementation requires understanding of the specific behavioural patterns and challenges faced by target populations.

### Aims of the Study

This study aimed to: (1) describe smoking status and physical activity patterns among Saudi patients following PCI; (2) explore associations between lifestyle factors (smoking and physical inactivity) and medication adherence, adjusting for clinical covariates; and (3) discuss implications for holistic cardiovascular nursing care addressing multiple secondary prevention behaviours.

## 2. Materials and Methods

### 2.1. Study Design

This study employed a cross-sectional quantitative design to characterise lifestyle behaviours and explore their associations with antiplatelet medication adherence among post-PCI patients in Saudi Arabia. The study is reported in accordance with the Strengthening the Reporting of Observational Studies in Epidemiology (STROBE) statement for cross-sectional studies [31].

### 2.2. Setting and Sample

Data were collected at two tertiary cardiac centres in Riyadh, Saudi Arabia. Both facilities are recognised as major cardiac centres offering advanced interventional cardiology services, including diagnostic catheterisation and PCI, and serve a broad catchment area encompassing both urban and semi-urban populations. Both centres maintain structured outpatient cardiology clinics. All data collection took place within the outpatient cardiology clinics of these two centres, where participants were attending routine follow-up appointments.

The target population comprised Saudi adults (≥18 years) who had undergone PCI within the preceding 12 months and were currently prescribed antiplatelet therapy (single or dual regimens). Patients with documented cognitive impairment, severe psychiatric illness, or inability to provide informed consent were excluded.

Consecutive patients presenting for routine cardiology follow-up during the recruitment period were approached by trained hospital-based research assistants who were not part of the authorship team. These assistants verified eligibility and explained the study objectives. Of 302 eligible patients approached, 66 were ineligible or declined to participate, and 236 consented and completed the survey, yielding a response rate of 78.1%.

### 2.3. Sample Size Considerations

The sample size was calculated using the formula for cross-sectional studies with a finite population, assuming an adherence prevalence of 50% based on international literature, a 95% confidence interval, and a 5% margin of error [32]. This yielded a minimum required sample of approximately 196 participants. With 236 participants enrolled, the sample exceeded this requirement for prevalence estimation.

A post hoc power consideration indicates that with 236 participants, a smoking prevalence of 23.3%, and an adherence prevalence of 44.6% in the reference group, the study had limited statistical power (estimated < 60%) to detect an odds ratio of 0.50 for the smoking–adherence association at α = 0.05.

We acknowledge that the total number of PCI procedures performed across the two centres during the recruitment window was not available to the research team; this information could not be retrieved from hospital administrative records within the scope of this study.

Data were collected using a structured questionnaire administered in a private consultation room within the outpatient cardiology clinic, away from other patients, with the option of self-completion or assisted completion for patients with limited literacy. On average, survey completion required 10–15 min. The full questionnaire is available from the corresponding author upon reasonable request; it was not included as a supplementary file due to journal word count restrictions. The questionnaire comprised four sections:

1. Demographic and Socioeconomic Questionnaire. Developed by the authorship team based on prior literature [33,34], this section gathered information on age (categorical), gender, marital status, education level, employment status, and monthly household income.
2. Clinical Health Profile. This self-reported section of the questionnaire was designed by the authorship team drawing on validated cardiovascular risk factor surveys and comprised questions about comorbidities (e.g., hypertension, diabetes mellitus, arthritis, dyslipidaemia, kidney disease, stroke), time since diagnosis of heart disease, and the number of PCI procedures undertaken. Data on PCI indication (e.g., STEMI, NSTEMI, stable coronary artery disease), stent type (drug-eluting vs. bare-metal), specific antiplatelet regimen, or medication cost were not collected, as the study relied on self-report rather than clinical record abstraction. This is acknowledged as a limitation (see Section 4.9).
3. Lifestyle Behaviour Assessment:
  - Smoking status: Participants were classified as non-smoker (encompassing both never-smokers and former smokers, as these were not distinguished in the original questionnaire) or current smoker. We note that this binary classification does not distinguish between never-smokers and former smokers; this is acknowledged as a limitation (see Section 4.9).
  - Cigarette consumption: For current smokers, daily cigarette consumption was categorised as ≤10, 11–20, 21–30, or >30 cigarettes per day.
  - Physical activity frequency: Assessed with a single item asking how often participants engaged in regular physical activity (e.g., walking, swimming, or other physical activity). Response options: never, once per week, twice per week, or ≥3 times per week. The item captures any self-reported physical activity and does not distinguish moderate-to-vigorous from light activity, nor does it capture duration or intensity. A brief single-item frequency question was selected to minimise participant burden in this older, clinically unwell population, in whom longer validated instruments such as the International Physical Activity Questionnaire (IPAQ) or Global Physical Activity Questionnaire (GPAQ) may result in fatigue-related response errors. This item was not drawn from a validated physical activity instrument and captures only frequency, not duration or intensity (see Strengths and Limitations, Section 4.9).
4. Morisky Medication Adherence Scale-8 (MMAS-8). This validated 8-item instrument assessed self-reported antiplatelet adherence [35]. The first seven items are dichotomous (yes/no), while the final item uses a 5-point Likert scale. Participants were classified as having high (score = 8), medium (score 6 ≤ 8), or low (score < 6) adherence. The Arabic MMAS-8 translation used is a pre-existing validated version, not one developed specifically for this study. Pilot testing with nine cardiac patients confirmed high comprehensibility, and Cronbach’s alpha in the study sample was 0.79, indicating good reliability. Written permission for use of the Arabic version of the MMAS-8 was obtained from the original author (Dr. Donald Morisky, who is not affiliated with the present research team, via a formal licensing agreement).

### 2.4. Data Analysis

Categorical variables are summarised as frequencies and percentages. Age data were collected using categorical age groups (≤50, 51–60, 61–70, 71–80, and >80 years) and are reported accordingly; age data were collected in categorical form to align with the study’s cross-sectional descriptive aim and because categorical age groups are commonly used in Saudi healthcare surveys. Continuous age data (mean and SD) were not collected and therefore cannot be reported.

For regression analysis, adherence was dichotomised as adherent (medium or high; MMAS-8 score ≥ 6) versus non-adherent (low; MMAS-8 score < 6). Bivariate associations between each independent variable and adherence were examined using chi-square tests (or Fisher’s exact test where expected cell counts were <5). Variables with *p* < 0.20 in bivariate analysis, along with clinically important factors, were entered into a multivariable binary logistic regression model to identify independent predictors of adherence. Age (categorical), sex, education, and income were not included in the final regression model because none met the *p* < 0.20 threshold for entry in bivariate analysis, and including them would have further reduced the events-per-variable ratio below the acceptable threshold. We acknowledge that socioeconomic determinants of adherence are well established; their exclusion represents an analytical limitation. Adjusted odds ratios (AORs) with 95% confidence intervals (CIs) are reported; a two-sided *p* < 0.05 was considered statistically significant. Given that six predictors were tested simultaneously, a Bonferroni-corrected significance threshold of *p* < 0.0083 (0.05/6) would apply if strict correction for multiple comparisons is adopted. Results are interpreted with this in mind (see Section 4.3). The events per variable (EPV) ratio was calculated to assess model stability. With 107 participants in the smaller outcome group (adherent: medium + high, n = 104; or non-adherent: n = 129) and 6 predictor variables in the model, the EPV ratio was approximately 17:1 (104/6), which exceeds the conventional threshold of 10:1 and suggests adequate model stability. The Hosmer–Lemeshow goodness-of-fit test yielded χ^2^ = 6.41, df = 8, and *p* = 0.60, indicating adequate model fit. Multicollinearity was assessed using variance inflation factors (VIFs); all VIF values were <2.0, indicating no problematic collinearity among predictors.

Income data were missing for 13.1% of participants (n = 31). Complete-case analysis was used; participants with missing income data were not excluded from overall sample descriptives, but income was not included as a covariate in regression models given the volume of missing data and its failure to reach the *p* < 0.20 entry threshold in bivariate analysis. Analyses were performed using IBM SPSS Statistics, Version 26.0 (IBM Corp., Armonk, NY, USA). All statistical tests were two-sided.

### 2.5. Ethical Considerations

The study was conducted in accordance with the Declaration of Helsinki. Ethical approval was obtained from the Institutional Review Board of King Saud University, Saudi Arabia, as well as from the Institutional Review Boards of the participating hospitals prior to data collection (IRB No. 17/0174/IRB and IRB No. 17-019E). All hospital protocols were strictly followed, and data collection commenced only after all required ethical approvals had been secured.

## 3. Results

### 3.1. Sociodemographic Characteristics

A total of 236 participants were enrolled in the study (Table 1). The sample was predominantly male (*n* = 174, 73.7%), married (*n* = 191, 80.9%), and had low educational attainment (33.1% no formal schooling; 45.3% primary and secondary [pre-university] education). The majority were older adults: only 16.1% (*n* = 38) were aged ≤50 years, while 25.8% (*n* = 61) were aged 51–60, 20.8% (*n* = 49) were aged 61–70, 25.4% (*n* = 60) were aged 71–80, and 11.9% (*n* = 28) were older than 80 years. Employment status was varied, with 31.4% retired, 26.7% currently employed, 24.2% unemployed, and 17.8% classified as housewives or disabled. The combination of ‘housewife’ and ‘disabled’ into a single employment category was a feature of the original data collection instrument; these groups cannot be retroactively separated and may have distinct implications for physical activity and health engagement (see Strengths and Limitations, Section 4.9). Household income was modest overall: 39.0% reported earning ≤ 4999 SAR per month (1 USD ≈ 3.75 SAR), while 13.1% had missing income data.

### 3.2. Clinical and Health Characteristics

Clinical comorbidities were highly prevalent (Table 2). Pre-existing heart disease was reported by 69.5% (n = 164) of participants—these are pre-existing cardiac conditions other than the index condition prompting PCI (e.g., prior myocardial infarction, heart failure, and valvular disease), as reported by participants—hypertension by 51.3% (*n* = 121), and diabetes mellitus by 45.0% (*n* = 106). Additional conditions included dyslipidaemia (24.1%, *n* = 57), arthritis (17.4%, *n* = 41), kidney disease (6.4%, *n* = 15), prior stroke (3.0%, *n* = 7), and other comorbidities such as respiratory or gastrointestinal disorders (7.6%, *n* = 18). The prevalence of arthritis (17.4%) in this cohort is consistent with the known co-occurrence of musculoskeletal and cardiovascular conditions in older adults sharing inflammatory and metabolic pathways; it is not unexpected given the predominantly older age distribution of this sample. Regarding disease history, 43.6% had been diagnosed with heart disease within the previous year, 17.9% for one to two years, 12.8% for three to four years, and 15.0% for five years or longer, while 10.7% were uncertain about their diagnosis duration. Most patients had undergone a single PCI procedure (62.3%), 20.8% had two procedures, and smaller proportions reported three (5.5%), four (1.7%), or five or more (2.1%) procedures; PCI frequency was unavailable for 6.8% of the sample.

### 3.3. Smoking Status and Patterns

The majority of participants (76.7%, n = 181) were classified as non-smokers (a category encompassing both never smokers and former smokers, as these groups were not distinguished in the questionnaire), while 23.3% (n = 55) identified as current smokers (Table 3). Among current smokers, cigarette consumption was predominantly low to moderate: 59.3% reported smoking ≤ 10 cigarettes daily. 25.7% smoked 11–20 cigarettes. 10.0% smoked 21–30 cigarettes, and 5.0% consumed more than 30 cigarettes per day.

### 3.4. Physical Activity Patterns

Physical inactivity was highly prevalent (Table 4). More than half of participants (57.2%, n = 135) reported never engaging in regular physical activity. Among those who were physically active, 21.2% (n = 50) reported exercising once per week, 11.9% (n = 28) twice per week, and 9.7% (n = 23) three or more times per week.

### 3.5. Medication Adherence Result

Adherence to antiplatelet therapy was suboptimal (Table 5). More than half of participants (55.4%, *n* = 129) demonstrated low adherence (MMAS-8 score < 6), 34.8% (*n* = 81) showed medium adherence (score 6 ≤ 8), and only 9.9% (*n* = 23) achieved high adherence (score = 8). For subsequent regression analysis, participants were dichotomised into adherent (medium + high; *n* = 104, 44.1%) versus non-adherent (low; *n* = 129, 54.7%); three participants with ambiguous scores were excluded, yielding an analytic sample consistent with the reported regression.

### 3.6. Continuity of Care and Medication Information Gaps

Continuity of care was moderate, with 58.5% (*n* = 138) routinely seeing the same cardiac specialist and 41.5% (*n* = 98) reporting follow-up with different specialists.

Experiences of medication-related information revealed important gaps (Table 6). Only 40.5% of participants reported always receiving clear explanations about their medications during hospital care, whereas 6.9% stated they never received such clarity. The greatest deficiency concerned side-effect communication: 26.4% (*n* = 61) reported that they had never been told about possible adverse effects, and only 26.0% (*n* = 60) reported always receiving this information. Understanding of medication instructions was somewhat better, with 46.0% always understanding instructions, although 14.0% acknowledged that they never understood them.

### 3.7. Multivariate Logistic Regression: Predictors of Adherence

In multivariable logistic regression analysis (Table 7), arthritis emerged as the only statistically significant predictor of adherence. Patients reporting arthritis were nearly three times more likely to be classified as adherent (medium/high vs. low) compared with those without arthritis (AOR = 2.81, 95% CI: 1.01–7.84, *p* = 0.048). However, this association does not survive Bonferroni correction for multiple comparisons (corrected threshold *p* < 0.0083) and should therefore be interpreted as a hypothesis-generating finding rather than a confirmed association. Hypertension was associated with more than double the odds of adherence (AOR = 2.41, 95% CI: 0.89–6.52, *p* = 0.084), although this association did not reach statistical significance.

Current smokers had approximately half the odds of being adherent compared with non-smokers (AOR = 0.52, 95% CI: 0.19–1.45, *p* = 0.213), but this was not statistically significant. Similarly, participants reporting regular physical activity showed reduced adherence odds (AOR = 0.45, 95% CI: 0.09–2.25, *p* = 0.332), also not reaching significance. Diabetes mellitus (AOR = 0.50, 95% CI: 0.19–1.33, *p* = 0.165) and heart disease (AOR = 0.71, 95% CI: 0.26–1.94, *p* = 0.505) were inversely but non-significantly related to adherence. The wide confidence intervals, particularly for physical inactivity (0.09–2.25), reflect the limited statistical power for these comparisons.

## 4. Discussion

### 4.1. Summary of Key Findings

This study characterised lifestyle behaviour patterns and explored their potential relationship with antiplatelet medication adherence among 236 Saudi post-PCI patients. Four key findings emerged: (1) nearly one-quarter of patients continued smoking after PCI; (2) over half reported no regular physical activity; (3) more than half demonstrated low medication adherence; and (4) arthritis was the only statistically significant predictor of higher adherence in multivariate analysis at the *p* < 0.05 threshold, though this finding is hypothesis-generating given that it does not survive correction for multiple comparisons, while smoking and physical inactivity showed inverse but non-significant trends.

### 4.2. Medication Adherence

The high prevalence of low adherence (55.4%) is consistent with prior reports of suboptimal antiplatelet adherence in Middle Eastern and global post-PCI populations. Studies conducted in the United States and Europe typically report adherence rates ranging from 70–80% during the first year after PCI [36,37], whereas research in Middle Eastern populations consistently demonstrates lower adherence, often below 50% [38,39]. The present findings are consistent with regional systematic reviews showing persistent challenges in medication management across Arab countries [40]. Contributing factors to lower adherence rates in the region include medication costs, health literacy gaps, cultural beliefs regarding fate, and reliance on family caregivers [27,28,41].

Particularly concerning were the gaps in medication education identified through the TPB-based questionnaire: 26.4% of patients had never received information about side effects, and 6.8% did not know which type of antiplatelet medication they were taking. These findings indicate failures in patient education and communication processes that have direct implications for nursing practice.

Our study did not collect data on the specific antiplatelet regimen prescribed (DAPT vs. SAPT, or specific agents), medication out-of-pocket cost, or insurance coverage. These are clinically important potential determinants of adherence. Differences in side effect profiles between agents, for example, dyspnoea with ticagrelor or gastrointestinal symptoms with aspirin, may differentially affect adherence without patients recognising or attributing these effects to their medication. The finding that 26.4% of patients never received information about side effects raises the important possibility that unrecognised or unexplained adverse effects may contribute to non-adherence. Future studies should document antiplatelet regimen type and cost burden as covariates.

### 4.3. Arthritis and Adherence

A notable finding was the association between comorbid arthritis and better adherence to antiplatelet therapy. Patients with arthritis were nearly three times more likely to be classified as adherent (AOR = 2.81, *p* = 0.048). However, several important caveats apply to this interpretation. The *p*-value is borderline; the confidence interval is wide (1.01–7.84), and the association does not survive Bonferroni correction for multiple comparisons (corrected threshold: *p* < 0.0083). Residual confounding by unmeasured variables (e.g., healthcare engagement frequency, polypharmacy routines, or pain-related health motivation) cannot be excluded. This finding should therefore be regarded as hypothesis-generating and treated with caution.

Several mechanisms could potentially explain the observed direction of this association if it were confirmed in adequately powered studies. Chronic musculoskeletal conditions often necessitate long-term engagement with healthcare services, which may enhance familiarity with medication routines and foster discipline in self-management [42]. Arthritis is associated with regular follow-up visits and pain management strategies, which may provide opportunities for reinforcing adherence behaviours [43]. Polypharmacy associated with arthritis management (e.g., NSAIDs, DMARDs) may establish structured medication-taking routines that generalise to antiplatelet therapy. Patients with multiple comorbidities sometimes demonstrate higher adherence because of increased perceived vulnerability and closer clinical monitoring [44]. The prevalence of arthritis in this cohort (17.4%) is consistent with the expected co-occurrence of musculoskeletal and cardiovascular conditions in an older adult population.

### 4.4. Hypertension and Adherence

Hypertension was associated with greater odds of adherence, though not statistically significant (AOR = 2.41, *p* = 0.084). This finding mirrors studies from Asia and North America where hypertensive patients often show better medication adherence due to the chronic nature of the condition and regular contact with healthcare professionals [45]. However, it also underscores the complexity of comorbidity–adherence relationships, as other studies have reported that multimorbidity may actually reduce adherence due to treatment burden and polypharmacy [46].

### 4.5. Smoking After PCI

The finding that 23.3% of participants continued smoking following PCI is clinically concerning, given that smoking cessation is among the most impactful secondary prevention interventions, substantially reducing cardiovascular morbidity and mortality [47]. This prevalence aligns with international literature documenting continued smoking rates of 20–40% among post-PCI patients [47,48,49]. Notably, our study did not distinguish between patients who had never smoked and those who had quit since their PCI; the questionnaire instrument used a binary current smoker/non-smoker classification, and data on pre-PCI smoking status or cessation were not collected. Future studies should capture pre-PCI smoking status to quantify successful cessation rates.

### 4.6. Physical Inactivity

Physical inactivity was particularly pronounced, with 57.2% of participants reporting no regular physical activity. This exceeds rates typically reported in Western cardiovascular populations and may reflect cultural, environmental, and health-system factors specific to Saudi Arabia, including limited access to appropriate exercise facilities, extreme climate conditions, and cultural norms regarding physical activity [50]. Physical activity was assessed with a single unvalidated categorical item; the absence of a validated instrument (e.g., the International Physical Activity Questionnaire [IPAQ]) limits the precision and comparability of this estimate. Moreover, the physical activity predictor entered into the regression model was derived from this single unvalidated frequency item, which limits the interpretability of the inactivity–adherence regression result as a valid test of the activity–adherence relationship. Cardiac rehabilitation attendance, the standard post-PCI exercise intervention—was not assessed in this study. Given that structured cardiac rehabilitation has demonstrated benefits for both physical outcomes and medication adherence [22,23], its non-assessment represents a significant gap that future studies should address.

### 4.7. Lifestyle–Adherence Associations

The inverse associations observed between smoking and physical inactivity with adherence were not statistically significant. Given the limited statistical power (<60%) for these comparisons, no directional conclusion can be drawn from the observed odds ratios. The wide confidence intervals (particularly for physical inactivity: 0.09–2.25) indicate substantial imprecision that precludes definitive conclusions about the presence, magnitude, or direction of these associations. Several plausible mechanisms could underlie a clustering of unhealthy behaviours with non-adherence if such a relationship were confirmed: shared psychological determinants (e.g., low self-efficacy and reduced health motivation) [51,52,53]; routine-based pathways (e.g., chaotic daily schedules among smokers) [54]; and differential healthcare engagement [55]. These mechanisms remain speculative in the context of the present study and warrant investigation in future studies designed to test them explicitly.

### 4.8. Implications for Nursing Practice and Future Research

Although the associations between lifestyle factors and adherence were not statistically significant (with the notable exception of arthritis), the descriptive findings, namely the high co-prevalence of multiple cardiovascular risk behaviours alongside substantial gaps in medication education, have practical relevance for nursing care planning. We offer the following observations, framed explicitly as hypothesis-generating propositions requiring confirmation in future adequately powered studies, rather than as practice recommendations supported by the present data:

1. Comprehensive behavioural assessment could be incorporated into routine post-PCI nursing care. Future research should test whether systematic evaluation of smoking status, physical activity levels, and medication adherence may enable identification of patients with multiple risk behaviours who could benefit from targeted intervention.
2. Structured medication education appears urgently needed based on the descriptive findings alone. The finding that 26.4% of patients never received information about side effects and 6.8% did not know their medication type highlights critical communication failures. Nurses, as the primary point of contact for patient education, are uniquely positioned to deliver structured, culturally tailored information at multiple time points along the care continuum.
3. Integrated counselling approaches that simultaneously address multiple behaviours represent a promising future strategy whose effectiveness should be tested in randomised trials. The PCI itself may represent a “teachable moment” during which patients are particularly receptive to health messages [56]. Motivational interviewing and other patient-centred counselling techniques may be valuable for patients exhibiting multiple risk behaviours [57].
4. Referral pathways to cardiac rehabilitation should be strengthened and their impact on adherence evaluated prospectively. Cardiac rehabilitation integrates supervised exercise, education, and psychosocial support, and has demonstrated benefits for multiple cardiovascular outcomes [58]. Uptake remains low in the Saudi context [59], and future research should quantify current cardiac rehabilitation referral and enrolment rates in this population.

These recommendations align with national healthcare transformation priorities under Saudi Vision 2030, which emphasise preventive care, patient engagement, and quality improvement. Future studies should also investigate whether prior SARS-CoV-2 infection affects lifestyle behaviours and medication adherence in post-PCI populations, given that a subset of COVID-19 survivors experience reduced exercise capacity and cardiovascular complications as part of long COVID sequelae. Such effects could confound associations between physical activity and adherence in studies conducted in populations with high prior COVID-19 exposure.

Future research should prioritise adequately powered prospective cohort studies employing validated measures of physical activity and objective adherence indicators (e.g., pharmacy refill records, electronic pill monitoring). Randomised controlled trials of integrated nurse-led interventions, particularly those grounded in behavioural frameworks such as the TPB, are needed to determine whether holistic approaches improve cardiovascular outcomes.

### 4.9. Strengths and Limitations

This study makes several contributions to the limited literature on lifestyle behaviours and medication adherence in Middle Eastern cardiovascular populations. The examination of multiple health behaviours within a single cohort provides a foundation for exploring clustering patterns that are novel in the Saudi post-PCI context. The multi-site recruitment from two major tertiary centres in Riyadh enhances the representativeness of findings within the urban Saudi cardiac care context. The acceptable response rate (78.1%), dual-site design, use of a validated adherence instrument (MMAS-8 with demonstrated reliability in this sample, α = 0.79), inclusion of both clinical and medication-education variables, and adherence to STROBE reporting guidelines enhance the credibility of the findings. The application of the TPB framework provides a theoretically grounded lens for interpreting adherence behaviours. Reporting of post hoc power considerations, EPV ratio, goodness-of-fit statistics, and multicollinearity diagnostics enhances methodological transparency.

Several limitations warrant consideration. First, the cross-sectional design precludes causal inference; temporality cannot be established. Second, all measures were self-reported, introducing potential recall and social desirability biases. Objective measures, cotinine testing for smoking verification, accelerometry for physical activity, and pharmacy refill records for adherence, would substantially strengthen future investigations. Third, the physical activity measure was a single unvalidated item that captured only frequency, not duration or intensity, limiting its construct validity and comparability with studies using standardised instruments such as the IPAQ or GPAQ; furthermore, the regression result for physical inactivity cannot be interpreted as a valid test of the activity–adherence relationship. Fourth, the study was likely underpowered to detect moderate associations between lifestyle factors and adherence; the wide confidence intervals for smoking and physical inactivity reflect this imprecision. Fifth, the dichotomisation of the MMAS-8 outcome involved combining medium and high adherence categories, which may have obscured a dose–response relationship. Sixth, age was collected as a categorical variable, precluding reporting of mean/SD and limiting its utility as a covariate. Seventh, the binary smoking classification does not distinguish never-smokers from former smokers, and potential confounders not measured, including health literacy, depression, polypharmacy burden, antiplatelet regimen type, medication cost, and type of antiplatelet regimen—may have influenced the observed associations. Eighth, clinical record linkage was not performed; accordingly, PCI indication, stent type, and specific antiplatelet regimen are unknown and could not be adjusted for in regression models. Ninth, the employment category combining ‘housewife’ and ‘disabled’ prevents separate analysis of these groups. Tenth, cardiac rehabilitation attendance was not assessed. Finally, the sample was drawn from two urban tertiary centres in Riyadh, potentially limiting generalisability to patients in rural settings, primary care, or other regions of Saudi Arabia. Qualitative insights into patients’ subjective experiences were not captured; future mixed-methods designs could provide more nuanced understanding. Additionally, prior SARS-CoV-2 infection, and associated long COVID effects on exercise capacity and cardiovascular health, was not assessed and may represent an unmeasured confounder in this population, given that data collection occurred in 2024.

## 5. Conclusions

This study documents a high prevalence of continued smoking (23.3% of the 236 participants; n = 55), physical inactivity (57.2% of the sample; n = 135), and low antiplatelet medication adherence (55.4% of the sample; n = 129) among Saudi post-PCI patients, alongside substantial gaps in medication education. Arthritis was the only predictor reaching conventional statistical significance in multivariate analysis, though this association is hypothesis-generating given its borderline *p*-value and failure to survive correction for multiple comparisons. Lifestyle factors (smoking, physical inactivity) showed inverse but non-significant trends with adherence; given the limited statistical power of the study, no directional conclusions can be drawn from these associations. The concurrent prevalence of multiple cardiovascular risk behaviours and medication education gaps in this cohort highlights an opportunity for integrated secondary prevention. Nurses are strategically positioned to address these gaps through structured, culturally sensitive counselling and follow-up. Future adequately powered prospective studies and trials of nurse-led integrated interventions addressing smoking cessation, physical activity promotion, medication education, and adherence support are needed to improve cardiovascular outcomes in this population.

## Figures and Tables

**Table 1:** Sociodemographic Characteristics of Participants (*N* = 236).

Variable	Category	*n*	%
Gender	Male	174	73.7
	Female	62	26.3
Marital status	Married	191	80.9
	Single	11	4.7
	Divorced	11	4.7
	Widowed	23	9.7
Education	No formal schooling	78	33.1
	Primary and secondary (pre-university)	107	45.3
	College diploma	43	18.2
	University or higher	8	3.4
Employment	Employed	63	26.7
	Unemployed	57	24.2
	Retired	74	31.4
	Other (housewife or disabled) *	42	17.8
Monthly income (SAR)	≤4999	92	39.0
	5000–7499	45	19.1
	7500–9999	17	7.2
	≥10,000	51	21.6
	Missing	31	13.1
Age group (years)	≤50	38	16.1
	51–60	61	25.8
	61–70	49	20.8
	71–80	60	25.4
	>80	28	11.9

* ‘Housewife’ and ‘disabled’ were combined in the original data collection instrument and cannot be separated retroactively.

**Table 2:** Clinical and Health Characteristics of Participants (N = 236).

Variable	Category	n	%
Comorbidities *	Heart disease †	164	69.5
	Hypertension	121	51.3
	Diabetes mellitus	106	45.0
	Arthritis	41	17.4
	Dyslipidaemia	57	24.1
	Kidney disease	15	6.4
	Stroke	7	3.0
	Other	18	7.6
Time since heart disease diagnosis	<1 year	102	43.6
	1–2 years	42	17.9
	3–4 years	30	12.8
	≥5 years	35	15.0
	Unknown	25	10.7
PCI history	One procedure	147	62.3
	Two procedures	49	20.8
	Three	13	5.5
	Four	4	1.7
	≥5	5	2.1
	Unknown	16	6.8

* Multiple responses allowed. † ‘Heart disease’ refers to pre-existing cardiac conditions other than the index condition prompting PCI (e.g., prior myocardial infarction, heart failure, valvular disease), as self-reported by participants.

**Table 3:** Smoking Status and Cigarette Consumption (N = 236).

Variable	Category	n	%
Smoking status	Non-smoker	181	76.7
	Current smoker	55	23.3
Cigarettes/day (n = 55)	≤10	33	59.3
	11–20	14	25.7
	21–30	5	10.0
	>30	3	5.0

**Table 4:** Physical Activity Frequency (N = 236).

Exercise Frequency	n	%
Never	135	57.2
Once per week	50	21.2
Twice per week	28	11.9
≥3 times per week	23	9.7
**Total**	**236**	**100.0**

**Table 5:** Antiplatelet Medication Adherence (MMAS-8 Categories, N = 236).

Adherence Level	MMAS-8 Score	*n*	%
Low	<6	129	55.4
Medium	6 ≤ 8	81	34.8
High	8	23	9.9

**Table 6:** Clarity of Medication Information Provided (N = 236).

Item	Never n (%)	Sometimes n (%)	Usually n (%)	Always n (%)
Clear explanation during hospital stay	16 (6.9)	68 (29.3)	54 (23.3)	94 (40.5)
Told purpose of new medication	26 (11.1)	60 (25.6)	56 (23.9)	92 (39.3)
Informed about side effects	61 (26.4)	65 (28.1)	45 (19.5)	60 (26.0)
Understanding medication instructions	33 (14.0)	94 (40.0)	–	108 (46.0)
Someone at home helps with medication	71 (30.2)	24 (10.2)	–	139 (59.1)

**Table 7:** Multivariate Logistic Regression: Predictors of Antiplatelet Adherence (Adherent vs. Non-Adherent).

Variable	Adjusted OR	95% CI	*p*-Value
Hypertension (yes vs. no)	2.41	0.89–6.52	0.084
Arthritis (yes vs. no)	2.81	1.01–7.84	0.048 †
Smoking (current vs. non-smoker)	0.52	0.19–1.45	0.213
No regular exercise (vs. any exercise)	0.45	0.09–2.25	0.332
Diabetes mellitus (yes vs. no)	0.50	0.19–1.33	0.165
Heart disease (yes vs. no)	0.71	0.26–1.94	0.505

† Statistically significant at *p* < 0.05 (conventional threshold); however, does not meet Bonferroni-corrected threshold of *p* < 0.0083 for six simultaneous comparisons. This finding should be interpreted as hypothesis-generating.

## Data Availability

The datasets generated and analysed during the current study are available from the corresponding author upon reasonable request. Due to participant privacy restrictions, data cannot be shared publicly.
